# Interleukin-32β Propagates Vascular Inflammation and Exacerbates Sepsis in a Mouse Model

**DOI:** 10.1371/journal.pone.0009458

**Published:** 2010-03-05

**Authors:** Hanako Kobayashi, Jianhua Huang, Fei Ye, Yu Shyr, Timothy S. Blackwell, P. Charles Lin

**Affiliations:** 1 Department of Radiation Oncology, Vanderbilt University Medical Center, Nashville, Tennessee, United States of America; 2 Department of Preventive Medicine, Vanderbilt University Medical Center, Nashville, Tennessee, United States of America; 3 Department of Medicine, Vanderbilt University Medical Center, Nashville, Tennessee, United States of America; 4 Department of Cancer Biology, Vanderbilt University Medical Center, Nashville, Tennessee, United States of America; 5 Department of Cell and Development Biology, Vanderbilt University Medical Center, Nashville, Tennessee, United States of America; Louisiana State University, United States of America

## Abstract

**Background:**

Inflammation is associated with most diseases, which makes understanding the mechanisms of inflammation vitally important.

**Methodology/Principal Findings:**

Here, we demonstrate a critical function of interleukin-32β (IL-32β) in vascular inflammation. IL-32β is present in tissues from humans, but is absent in rodents. We found that the gene is highly expressed in endothelial cells. Three isoforms of IL-32, named IL-32α, β, and ε, were cloned from human endothelial cells, with IL-32β being the major isoform. Pro-inflammatory cytokines (TNFα and IL-1β) induced IL-32β expression through NF-κB. Conversely, IL-32β propagated vascular inflammation via induction of vascular cell adhesion molecules and inflammatory cytokines. Accordingly, IL-32β increased adhesion of inflammatory cells to activated endothelial cells, a paramount process in inflammation. These results illustrate a positive feedback regulation that intensifies and prolongs inflammation. Importantly, endothelial/hematopoietic expression of IL-32β in transgenic mice elevated inflammation and worsened sepsis. This was demonstrated by significant elevation of leukocyte infiltration and serum levels of TNFα and IL-1β, increased vascular permeability and lung damage, and accelerated animal death. Together, our results reveal an important function of IL-32 in vascular inflammation and sepsis development.

**Conclusions/Significance:**

Our results reveal an important function of IL-32 in vascular inflammation and sepsis development.

## Introduction

Inflammation is a protective mechanism elicited by the host in response to infection, injury, and tissue damage. It is closely associated with the development and progression of a variety of diseases. Vascular endothelium is an active participant in inflammation [1], [2]. Inflamed endothelium mediates diverse activities that include the regulation of leukocyte recruitment and infiltration, cytokine production, and vascular permeability [3]. During inflammation initiation, circulating leukocytes must first be able to adhere selectively and efficiently to vascular endothelium. This process is facilitated by induction of vascular cell adhesion molecules on the inflamed endothelium, such as vascular cell adhesion molecule (VCAM)-1, intercellular adhesion molecule (ICAM)-1, and E-selectin [1], [2]. It is evident that the endothelium is a critical gatekeeper that controls the recruitment of distinct leukocyte subpopulations and, in doing so, determines the nature and extent of acute and chronic inflammation. Endothelium also exerts a potent inflammatory role by secreting inflammatory cytokines, such as IL-1 and TNFα, which upregulate ICAM-1 expression and enhance leukocyte adherence to the activated endothelium [1], [2].

NK4 was originally isolated from activated human natural killer cells [4]. Its expression is increased upon activation of NK cells by IL-2 or activation of T cells by mitogens. This gene was rediscovered in human lymphocytes, and renamed IL-32 [5]. Although IL-32 does not share sequence homology with any known cytokine families, IL-32 induces various cytokines, such as TNFα and IL-8 in lymphocytes and monocytic cells [5]. The full length IL-32 gene is composed of 705 base pairs. IL-32 exists as four splice variants in blood cells, named IL-32α, β, γ and δ, with IL-32α as the major isoform in hematopoietic cells [5]. The highest homology to human IL-32 was found in equine tissue at 31.8%, and no homologue to this gene was found in mice. Since IL-32 expression is regulated by inflammatory cytokines in human peripheral lymphocyte cells, it has been speculated that it may play a role in inflammatory/autoimmune diseases [5]. Further analysis indeed showed an elevation of IL-32 in human inflammatory diseases, such as in rheumatoid arthritis [6], [7], ulcerative colitis and Crohn's disease [8].

Here, we report a new function of IL-32 in vascular inflammation and sepsis development. We found that IL-32β is highly expressed in human endothelial cells. IL-32β amplifies and sensitizes vascular inflammation through induction of inflammatory cytokines and cell adhesion molecules, and subsequently leukocyte adhesion. Importantly, endothelial/hematopoietic expression of human IL-32β in transgenic mice elevated inflammation and worsened sepsis. Thus, this study identifies a critical mediator in vascular inflammation and sepsis progression.

## Methods

### Ethics

All animal studies have been conducted according to Animal Welfare Act and the Public Health Service Policy. The animals were housed in pathogen-free units at Vanderbilt University Medical Center, in compliance with IACUC regulations.

### Cell Culture

Human umbilical vein endothelial cells (HUVECs) and human coronary artery endothelial cells (HCAECs), and human aortic smooth muscle cells (HASMCs) were purchased from Clonetics (San Diego, CA). Human breast epithelial cells (MCF-7), human colon epithelial cells (HT29), human lung epithelial cells (H460), human glioma cells (U251MG), human prostate epithelial cells (DU145), human embryonic kidney epithelial cells (HEK293), human fibroblast cells (CCD21-SK), human myeloblastic cells (HL60), and human monocytes (THP-1) were purchased from ATCC. Adenoviral vectors directing the expression of IκB (AdIκBα), a mutated IκB as a NF-κB inhibitor, and GFP (AdGFP) as viral vector controls were used [9], [10].

### IL-32 cDNA Synthesis and Cloning

The cDNAs were synthesized from cultured HUVECs. The cDNAs were used to amplify IL-32 using the specific primer set: GGGAATTCATGTGCTTCCCGAAGGTC and GGCTCGAGTCATTTTGAGGATTGGGG. PCR products were cloned into pAdTrack-CMV and packaged with pAdEasy-1 for adenovirus preparation (AdIL-32β).

### Northern Blot

Cells were infected with adenoviral vectors expressing genes of interest for 48 hour. Cells were then serum starved for 5 hours, followed by treatment with IL-1b or TNF-α (R D Systems) at 10 ng/ml. RNA was subjected to Northern blot analysis. ^32^P labeled cDNA probes for IL-32 mRNA were hybridized using Express Hyb (BD Biosciences).

### q RT-PCR

Total RNA was isolated from HUVECs infected with either AdGFP or AdIL-32β for 48 hours and from mouse lung endothelial cells and BM. The primer sets are: ICAM-1 CCACAGTCACCTATGGCAAC/AGTGTCTCCTGGCTCTGGTT; VCAM-1 GCTTCAGGAGCTGAATACCC/AAGGATCACGACCATCTTCC; E-selectin TGAACCCAACAATAGGCAAA/CCTCTCATCATTCCACATGC, IL-1β CCCAACTGGTACATCAGCAC/GGAAGACACAAATTGCATGG, TNFα CCTCTTCTCCTTCCTGATCG/ATCACTCCAAAGTGCAGCAG, and IL-32 CGACTTCAAAGAGGGCTACC/GAGTGAGCTCTGGGTGCTG. Relative levels of IL-32 mRNA in IL-32 transgenic mouse was calculated by subtracting the wild type value from the value in the transgenic mouse. We assume the reading in wild type mouse as zero since IL-32 is absent in rodent.

### Transfection

HUVECs were transfected with IL32 shRNA target plasmid set (Sigma-Aldrich) using transfection kit (Amaxa). One shRNA (#59213) in the target set does not target any genes, and it was used as a control.

### Cell Adhesion Assay

HUVECs were infected with either AdGFP or AdIL-32β, then treated with recombinant IL-1β for 4 hours. THP-1 cells were labeled with PKH26 Red Fluorecent Cell Liner kit (Sigma-Aldrich) and allowed to adhere to the monolayer of HUVECs for 30 min. Adhered THP-1 cells were counted under fluorescent microscopy. Similarly, microvascular endothelial cells were isolated from mouse lungs as described [11], and subjected to cell adhesion assay.

### IL-32 Transgenic Mice

We constructed a plasmid in which IL-32β human transgene is driven by an endothelial/hematopoietic specific, platelet endothelial cell adhesion molecule-1 (PECAM-1) promoter, which was kindly provided by Dr. HS Baldwin at Vanderbilt. Using the plasmid, a transgenic mouse line was generated in FVB background at the Vanderbilt Transgenic Mouse Core.

### CLP Model

Ten weeks old mice were used for the cecal-ligation and puncture (CLP) model as described [12]. Seventeen hours after CLP, mice were sacrificed and blood was drawn. Serum concentrations of IL-1β and TNFα were measured using commercial ELISA kits (R & D system). Lung tissues were harvested and embedded in paraffin. Histological sections were stained with H&E.

A dorsal skin window was established in the back skin fold of a mouse as described for 24 hours [13], followed by the CLP procedure. Six hours after the CLP, 1 mg of dextran tetramethylrhodamine (10,000 MW) in 100 ul saline was injected via tail vein. Vasculature was imaged through the window chamber using fluorescent microscopy.

### Statistics

Results are reported as mean ± SEM. Statistical analysis was done using a two-tailed Student's t-test. Differences were considered statistically significant at p<0.05. Sepsis study was graphed in Kaplan-Meier format and analyzed by a log-rank test.

## Results

### IL-32 Is Highly Expressed in Vascular Endothelial Cells

In searching for Akt target genes in human endothelial cells, we identified NK4, which was recently renamed IL-32 [5]. Sequence analysis identified three isoforms of IL-32, which are 489 bp (IL-32α); 567 bp (IL-32β); and 507 bp (IL-32ε). Among them, IL-32β is the major isoform in endothelial cells.

**Figure 1:**
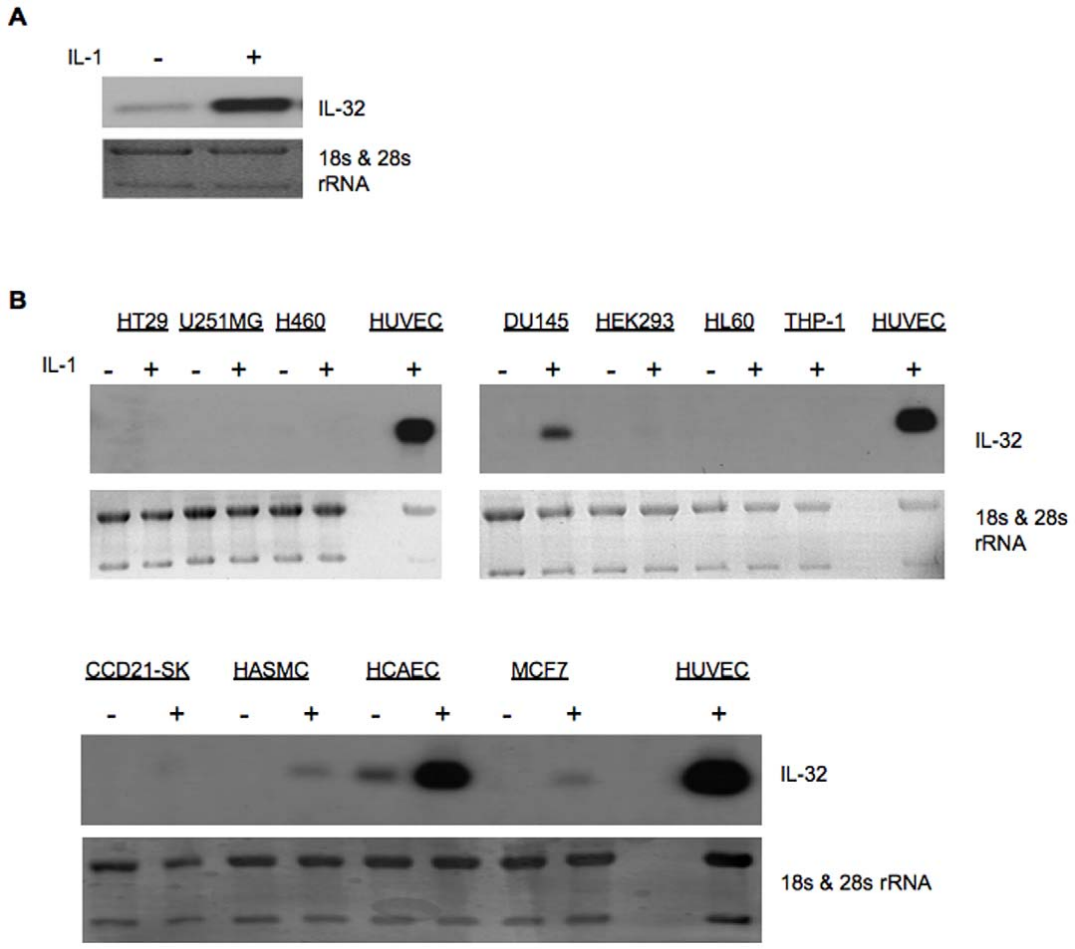
IL-32 is highly expressed in vascular endothelial cells. HUVECs were stimulated with or without IL-1β at 10 ng/ml for 24 hours. Total RNA were isolated and analyzed by Northern blot for IL-32 (Panel A). Cells were treated with or without IL-1β at 10 ng/ml for 24 hours. Total RNAs isolated from different types of human cells were analyzed by Northern blot for IL-32 (Panel B). 18S & 28S rRNA were used as a loading control.

As IL-32 is reportedly ubiquitously expressed in human tissues [5], we next compared the expression of IL-32 in different types of human cells, including endothelial cells, epithelial cells, smooth muscle cells, fibroblast cells and leukocytes. Interestingly, we found a high level expression of IL-32 in endothelial cells, including large and small vascular endothelial cells, with minimum or undetectable levels in most non-endothelial cells (Figure 1).

### IL-1β and TNFα Induce IL-32β Expression in an NF-κB Dependent Manner

**Figure 2:**
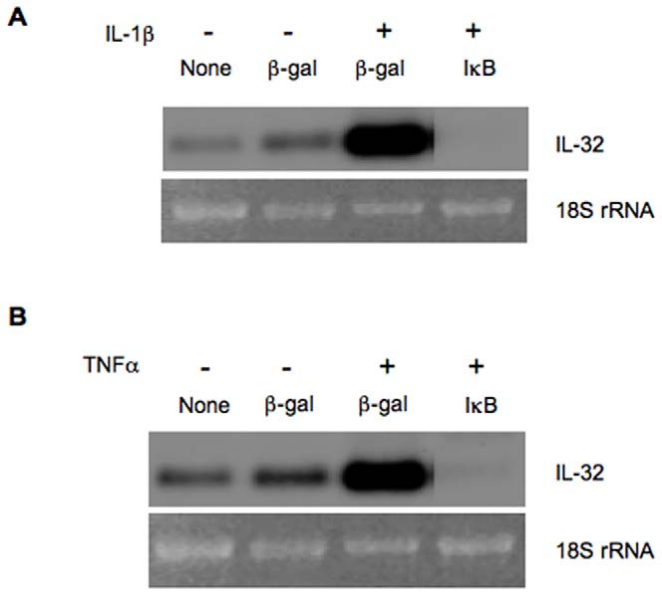
Inflammatory cytokines induce IL-32 expression via NF-κB. HUVECs were infected with control Adβ-gal or AdIκB adenovirus (MOI = 10) for 36 hours, followed by incubation with recombinant IL-1β (Panel A) or TNFα (Panel B) at 10 ng/ml for 12 hours. Uninfected cells were used as a control (none). IL-32 mRNA was analyzed by Northern blot. The experiments were repeated at least 3 times.

To investigate the function of IL-32β in endothelial biology, we studied gene regulation. We found that inflammatory cytokines dramatically induced IL-32β expression in cultured human endothelial cells (Figure 1, Figure 2A and 2B), suggesting a role of IL-32β in vascular inflammation. As NF-κB is a master mediator that regulates the expression of a variety of genes involved in inflammation; we examined its role in IL-32β gene expression. We found that NF-κB activity is essential for IL-32β expression in response to inflammatory cytokine stimulation. Blocking NF-κB function using a mutant IκB [9] completely abolished TNFα- and IL-1-induced IL-32β expression in endothelial cells (Figure 2A and 2B). Furthermore, NF-κB activity is also required for basal level expression of IL-32β, as there is no detectable amount of the gene in mutant IκB treated group compared to noticeable expression in unstimulated cells.

### IL-32β Amplifies Vascular Inflammation via the Production of Inflammatory Cytokines and Vascular Cell Adhesion Molecules, and Subsequent Adhesion of Leukocytes

**Figure 3:**
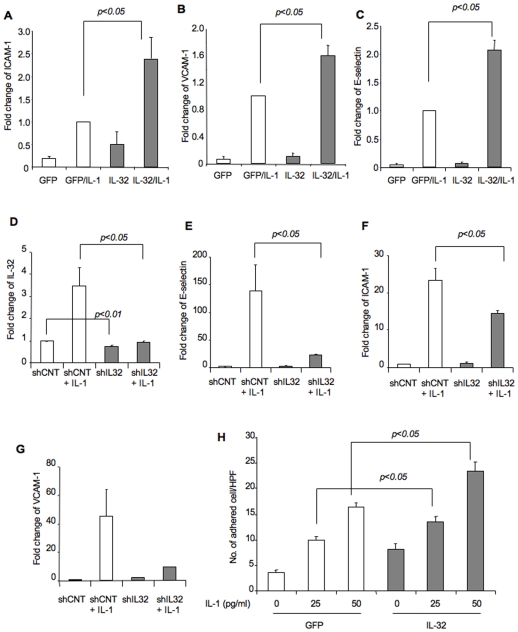
IL-32β increases the expression of vascular cell adhesion molecules and subsequent leukocyte adhesion. HUVECs were infected with either AdIL-32β or AdGFP vector for 48 hours, and then treated with IL-1β at 100 pg/ml for 4 hours. qRT-PCR was performed for the indicated cell adhesion molecules. Each gene was normalized with β-actin. Fold change of gene expression was calculated against the GFP/IL-1 (Panel A-C). HUVECs were transfected with IL32 shRNA target plasmid or control shRNA plasmid, followed by treatment with IL-1β for 48 hours. qRT-PCR was performed for the indicated IL-32 and cell adhesion molecules. Fold change of gene expression was calculated against the shCNT treated group (Panel D-G). HUVECs were infected with either control AdGFP or AdIL-32β for 36 hours, followed by incubation with IL-1β at the dose indicated for 4 hours. THP-1 leukocyte adhesion was measured 30 minutes after incubation and counted under microscopy (Panel H). All the experiments were repeated at least 3 times. The data are presented as mean ± SEM. *p<0.05.

Next, we investigated the molecular mechanism of IL-32β in vascular inflammation. The expression of cell adhesion molecules, such as ICAM-1, VCAM-1, and E-selectin, on activated endothelium is an initial and critical event in inflammation. We found that expression of IL-32β (5.4 ± 0.1 fold increase over GFP control) alone had no effects on the expression of cell adhesion molecules. However, it significantly increased cellular responses to a very low dose of IL-1β stimulation (Figure 3A, 3B and 3C). Further analysis revealed that the level of IL-32 mRNA is approximately 3.1 fold and 11.3 fold higher in IL-1β stimulated control vector transfected and IL-32β vector transfected cells than corresponding non-stimulated cells, respectively. Although we only achieved 25.9 ± 3.5% knockdown of basal levels IL-32, the reduction was significant and most importantly it totally blocked IL-1 induced IL-32 gene expression (Figure 3D). Consistently, blocking IL-32 induction in endothelial cells using shRNA significantly attenuated IL-1β induced expression of cell adhesion molecules (Figure 3E, 3F and 3G). Accordingly, an analysis of leukocyte adhesion on stimulated endothelial cells demonstrated that significantly increase of leukocytes adhered to IL-32β transfected endothelial cells (Figure 3H). These data suggest that IL-32β sensitizes endothelial cells and/or amplifies vascular inflammation through elevated expression of cell adhesion molecules and subsequent leukocyte adhesion on activated vascular endothelium.

In addition to cell adhesion molecule expression, endothelium is also a major source of cytokine production during inflammation. One of the interesting features of pro-inflammatory cytokines is that they induce their own expression, and thus propagate and prolong inflammatory signals. To test this role of IL-32β, we transfected HUVECs with either an IL-32β expression vector or an empty vector for 24 hours, followed by stimulation with a sub dose of IL-1β. We detected a 2.2 fold (95% CI; 1.1, 4.7) increase in IL-1β mRNA levels in IL-32β vector transfected cells compared to controls. Collectively, these data suggest a positive feedback mechanism, in which inflammatory cytokines induce IL-32β expression, and IL-32β further amplifies cytokine-induced inflammatory responses through increased leukocyte adhesion, as well as increased production of pro-inflammatory cytokines. As a result, inflammation may be intensified.

### IL-32β Promotes Inflammation and Exacerbates Sepsis In Vivo

**Figure 4:**
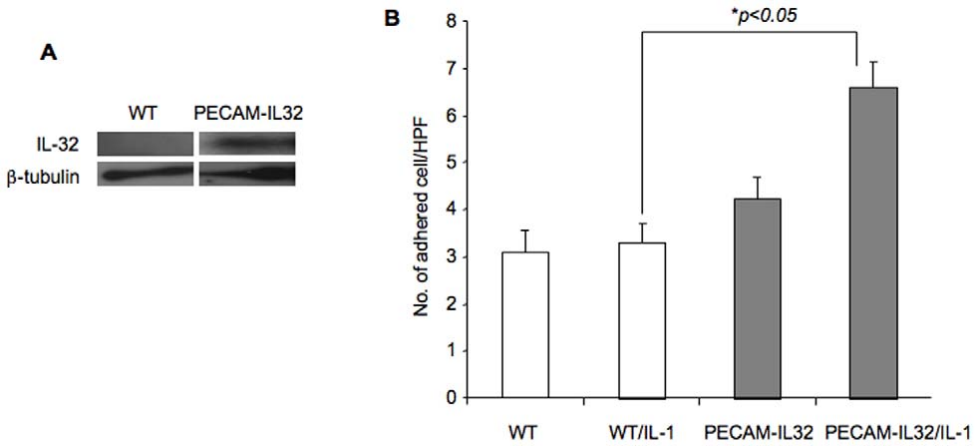
Characterization of PECAM-IL32 transgenic mice. Microvascular endothelial cells were isolated from lungs of PECAM-IL32 mice and wild type littermates. The level of IL-32β expression was determined Western blot (Panel A). Freshly isolated lung vascular endothelial cells from multiple wild type or PECAM-IL32 mice were treated with IL-1β at 100 pg/ml or vehicle control for 4 hours. THP-1 leukocyte adhesion was measured 30 minutes after incubation and counted under microscopy (Panel B). The experiment was repeated at least three times. *p<0.05.

One of the interesting and unique features of IL-32 is its absence in mice. To further characterize the function of IL-32β in inflammation *in vivo*, we generated a transgenic mouse line (PECAM-IL32), in which IL-32β is driven by an endothelial/hematopoietic cell specific promoter, the PECAM-1 promoter [14]. PECAM-IL32 transgenic mice are grossly healthy, viable and fertile. Vascular endothelial transgene expression was verified using microvascular endothelial cells isolated from lungs of transgenic mice (Figure 4A). Accordingly, there is a significant increase in leukocyte adhesion on lung endothelial cells isolated from the transgenic mice compared to those from wild type littermates (Figure 4B), consistent with the *in vitro* data and supporting a role of IL-32β in vascular inflammation.

**Figure 5:**
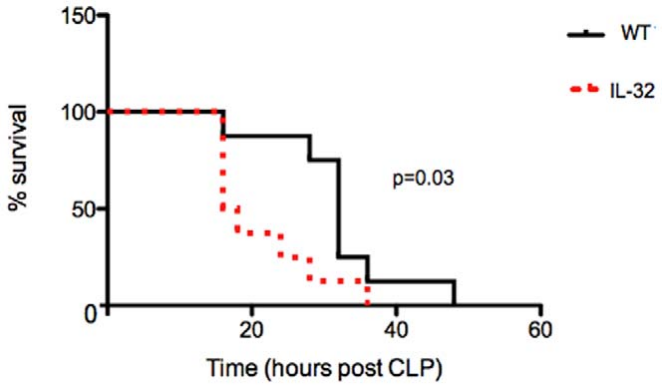
Over-expression of IL-32β *in vivo* accelerates animal death under septic conditions. The cecal-ligation and puncture model was used to induce sepsis in wild type and PECAM-IL32 mice. Survival curve is presented. n = 12 for each group.

**Figure 6:**
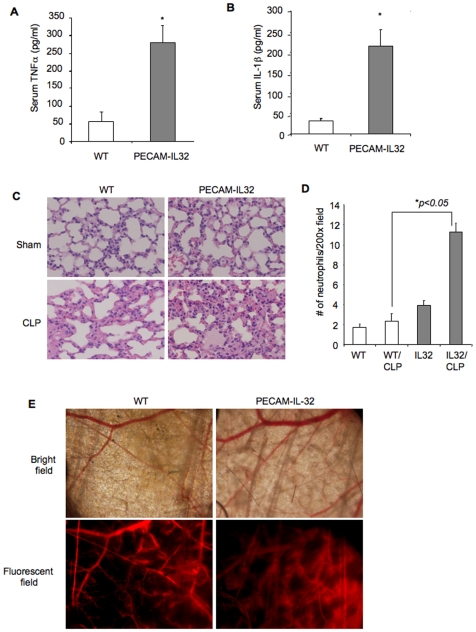
Over-expression of IL-32β *in vivo* increases inflammation and exacerbates sepsis. Seventeen hours after the CLP procedure, TNFα and IL-1β levels in serum were measured by ELISA. *p<0.05. n = 5 mice for each group (Panel A and B). At the same time, lung tissues sections were stained with H&E. Representative images (200X) are shown (Panel C). Infiltrating neutrophils were counted in ten randomly selected high power fields (200X) and graphed (Panel D). n = 5 mice per group, *p<0.05. A vascular window chamber was installed on the back skin fold of mouse for 24 hours, followed by the CLP procedure or sham operation. Dextran tetramethylrhodamine (10,000 MW) at 1 mg in 100 ul saline was injected via tail vein 6 hours after the CLP procedure. Vasculature was imaged through the window chamber under a fluorescent microscope (Panel E). Representative images were shown. n = 4 mice per group.

Sepsis is a systemic inflammatory response to bacterial infection. Widespread inflammation causes rapid changes in body temperature, blood pressure, and vascular permeability, and dysfunction in the lungs and other organs, often leading to death. Thus, we tested the function of IL-32β in inflammation using a cecal-ligation and puncture (CLP) sepsis model. As expected, animal death was significantly accelerated in PECAM-IL32 mice compared to wild type mice (p = 0.03) (Figure 5). Consistently, we observed a dramatic increase of TNFα and IL-1β serum levels in the IL-32 transgenic mice (p<0.01) (Figure 6A and 6B), a significant increase in inflammatory cell infiltration, edema, thickening of the alveolar wall and severe tissue damage in lungs of the PECAM-IL32 mice compared to the ones from the wild type mice (Figure 6C). We also found significantly higher levels of neutrophil infiltration in lungs of PECAM-IL-32 mice than in wild type controls (Figure 6D). These phenotypes are consistent with the clinical observations in septic patients.

Increased vascular leakiness is a hallmark of sepsis. To examine the effects of IL-32β on vascular permeability during sepsis development, we placed a vascular window chamber in the back skin fold of a mouse prior to the CLP procedure. Six hours after the CLP, rhodamine-dextran conjugate was intravenously injected, and vascular leakiness was directly imaged in live mice. There is a clear increase in blood vessel dilation and vascular leakiness in IL-32 transgenic mice compared to wild type controls (Figure 6E). Together with the *in vitro* studies, our findings reveal a positive role of IL-32β in vascular inflammation. IL-32β amplifies and propagates inflammatory reactions that worsen sepsis and lead to animal death.

## Discussion

**Figure 7:**
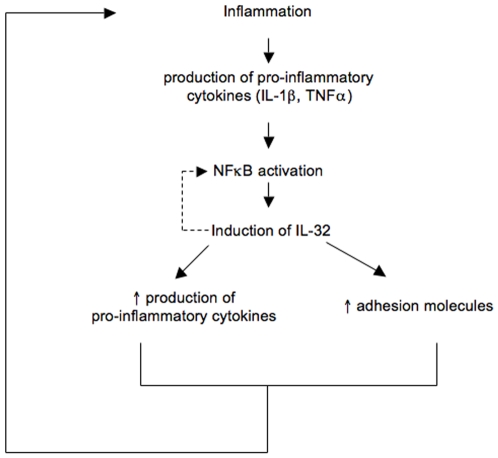
Diagram of positive feedback regulation of IL-32β in vascular inflammation.

Vascular inflammation plays a critical role in pathological conditions [1], [2], [3]. In this study, we identify a new function of IL-32β in vascular inflammation. We show that IL-32β is highly expressed in vascular endothelial cells, and pro-inflammatory cytokines upregulate its expression in a NF-κB dependent manner. This finding is consistent with a recent publication in endothelial cells [15]. IL-32β sensitizes/amplifies inflammatory cytokine mediated expression of cell adhesion molecules, as well as the expression of those same cytokines. Accordingly, it enhances leukocyte adhesion on inflamed endothelium. These data illustrate a positive feedback mechanism, in which inflammatory cytokines induce IL-32β expression, and subsequently IL-32β increases cellular responses to propagate the expression of cellular adhesion molecules and adhesion of leukocytes to inflamed endothelium, as well as pro-inflammatory cytokines. As a result, inflammation is amplified and prolonged (Figure 7). Interestingly, one study has demonstrated an IL-32-mediated degradation of IκB in lymphocytes [5], which could add another level to the positive regulation of inflammatory responses. Most importantly, endothelial/hematopoietic expression of human IL-32β in transgenic mice intensified inflammation and exacerbated sepsis. Considering inflammation is a risk factor for a variety of diseases, this study could have important implications in sepsis and other diseases.

One of the features of IL-32 that drew our attention is the fact that IL-32 does not exist in the rodent genome or published genomic libraries of other species. A similar search performed for IL-32 using the NCBI databank showed the highest homology (31.8%) with human in equine IL-32 (AC: CD469554 and BI961524) [5]. It was followed by bovine (AC: CK832489), ovine (AC: CO202364), and swine (AC: CB287292). These findings led us to examine gene expression, and we found that IL-32 is expressed in humans and certain primates (data not shown). This result suggests IL-32 may be a “human/primate specific gene”. Interestingly, mice are commonly used as model systems to study human diseases. However, we often observe reduced or attenuated inflammatory responses in rodent disease models compared to human conditions. Therefore, an understanding of the differences at the genetic level between us and rodents could have major implications on our health.

An analysis of B cells from 18 individuals revealed variable banding patterns suggestive of polymorphism within the NK4 gene [4]. In this study, we cloned three different spliced isoforms of IL-32 in endothelial cells, IL-32α, IL-32β and IL-32ε, with IL-32β being the dominant isoform in endothelial cells. Recently, several isoforms of IL-32 were reported in lymphocytes, which include IL-32α, IL-32β, and IL-32γ (full length), and IL-32δ in NK cells [5], as well as IL-32β, IL-32δ, IL-32ε, and IL-32ζ in T cells [16]. IL-32α was shown to be the major isoform in NK cells [5], whereas IL-32β was predominant in T cells [16] and endothelial cells. Nold-Petry et al showed a switch of isoforms from IL-32α and IL-32γ to IL-32β and IL-32ε to some degree in HUVECs upon IL-1β treatment [15]. These findings indicate that IL-32 is processed differently in different types of cells, and different isoforms may function differently in each cell type. It would be interesting to determine the functions of each isoform in each type of cells.

The vascular network, being the interface of two compartments (blood and tissues), plays a key role in inflammation. The interaction of inflammatory cells with inflamed endothelium is an initial and paramount process in inflammation. In this study, we show that IL-32β amplifies inflammatory cytokine mediated vascular inflammation. IL-32β alone has no or minimal effects on the expression of cell adhesion molecules and pro-inflammatory cytokines in endothelium. However, IL-32β enhances the cellular response to very low dose of cytokine stimulation, which results in enhanced leukocyte adhesion. These results imply that IL-32β itself is not a cytokine, but it amplifies and sensitizes the cellular response to cytokine stimulation. This result is consistent with our recent publication, in which we showed that IL-32b is not a secreted protein rather an intracellular protein localized in the ER [17].

To investigate the function of IL-32β *in vivo*, we generated a transgenic mouse line using the PECAM-1 promoter. PECAM-1 is only expressed in endothelium and hematopoietic cells [14], [18]. PECAM-1 promoter has been used to express transgene in endothelial/hematopoietic cells. Although using this promoter may not allow us to pin point the direct contribution of endothelial IL-32 in vascular inflammation *in vivo*, but it is also advantageous, simply because it reflects high levels endogenous IL-32 expression in endothelial and hematopoietic cells. There are a few other promoters that are deemed “endothelial specific”, such as the Tie2 promoter. However, emerging data suggest Tie2 is also expressed in hematopoietic cells, such as monocytes [19]. The same story goes for the VEGF receptor 2, another so called “endothelial specific” gene. These expression patterns reflect a common origin between hematopoietic cells and endothelial cells.

Sepsis is a systemic inflammatory reaction, and severe sepsis often leads to death. The cause of sepsis is not the infection itself, but rather the host inflammatory response. Endothelium dysfunction is one of the hallmarks of sepsis [20]. When production of the inflammatory mediators and phenotypic alterations are uncontrolled and excessive, this leads to vascular and tissue damage, organ dysfunction and death. Consistent with the *in vitro* data, we demonstrate that endothelial/hematopoietic expression of human IL-32β in mice increased inflammatory cell infiltration, vascular leakiness and tissue damage in lungs that resulted in a significant acceleration of animal death compared to wild type control. Serum TNFα and IL-1β levels, two primary mediators of septic shock, are significantly increased in these mice as well. These results suggest IL-32β as a positive mediator in sepsis development. In addition to our findings on vascular inflammation and sepsis, IL-32 has been implicated in inflammatory diseases. Injection of recombinant human IL-32γ in mouse knee joints caused rheumatoid arthritis-like symptoms [6], and overexpression of human IL-32β mouse bone marrow cells exacerbated collagen-induced arthritis and trinitrobenzen sulfonic acid-induced colitis [7].

In summary, this study has identified a critical function of IL-32β in vascular inflammation and sepsis development. Considering the close association of inflammation with human disease, and that IL-32 seems to be a “human/primate specific gene”, understanding the function of IL-32 could have a significant impact on our health. Of additional note, the IL-32β transgenic mice may offer a better and more relevant system for studying human diseases, as we often observe partial and reduced phenotypes in rodent disease models compared to humans.
